# Headache in children

**DOI:** 10.1186/1129-2377-14-S1-O3

**Published:** 2013-02-21

**Authors:** Prab Prabhakar

**Affiliations:** 1 Great Ormond Street Hospital, UK

## 

One in 30 children who attend primary school have a headache problem which has an impact on the child and family. However, the problem is under recognised and under treated. In children, identifying a primary headache syndrome can be challengining. However, it can be achieved in a focussed consultation undertaking appropriate history and examination. The course aims to cover the common pitfalls and red flags in the diagnosis of primary headache disorders in childhood and treatment startegies.

